# Bridging the language gap in palliative oncology: A translation and validation of the Urdu version of the EORTC QLQ-C15-PAL

**DOI:** 10.1017/S1478951525101521

**Published:** 2026-01-21

**Authors:** M. Abdullah Jamil, Syed Balaj Ali Rizvi, Aisha Ambreen, Asra Taj, Ismat Jabeen, Habiba Zaheer, Hunza Asher, Mahnoor Javed, Omar Mahmud, Muhammad Atif Waqar

**Affiliations:** 1 Medical College, Aga Khan University, Karachi, Pakistan; 2 Section of Palliative Medicine, Department of Oncology, Aga Khan University Hospital, Karachi, Pakistan

**Keywords:** Quality of life, palliative care, Urdu, EORTC QLQ-C15-PAL, patient-reported outcomes

## Abstract

**Objectives:**

The European Organization for Research and Treatment of Cancer Quality of Life Questionnaire Core 15 Palliative Care (EORTC QLQ-C15-PAL) is designed to measure quality of life (QoL) in cancer patients receiving palliative care. The aim of this study was to translate and validate an Urdu version of the questionnaire, which was previously lacking.

**Methods:**

Following formal approval from the EORTC, the QLQ-C15-PAL was translated into Urdu. Patients admitted under the palliative care service at a tertiary care center in Karachi, Pakistan, were enrolled in this cross-sectional study, and the Urdu QLQ-C15-PAL and the Edmonton Symptom Assessment Scale (ESAS) forms were administered. Performance status was assessed using the Palliative Performance Scale (PPS). Cronbach’s alpha and Pearson correlation coefficients were determined to gauge reliability and validity. Concurrent and known-group validity were tested using ESAS responses and PPS assessments.

**Results:**

One hundred patients with varying primary cancer sites were included. Cronbach’s alpha for the overall questionnaire was 0.86 and was >0.8 for all subscales except fatigue, where it was 0.697. All correlations to indicate convergent validity had coefficients >0.8 and 87% of correlations between “unrelated” domains were weak, indicating discriminant validity. Known group validity was established and improved QoL was observed in the high PPS (>40) subgroup of patients across multiple domains. However, concurrent validity was not strongly established.

**Significance of results:**

The Urdu QLQ-C15-PAL is a reliable and valid tool to measure QoL in cancer patients who speak Urdu. However, replication of our results in other settings is warranted.

## Introduction

The term “palliative care” was coined in the 1970s to avoid the negative connotation of “hospice.” Palliative care began with a focus on the care of the dying and is usually defined as a holistic approach that aims to improve the quality of life (QoL) of patients suffering from life-threatening illnesses and their families (Loscalzo 2008; Palliative care 2020).

The World Health Organization estimates that around 56.8 million people require palliative care each year, and only 7.9 million of them receive it (Palliative care 2020). For perspective, there were an estimated 18.1 million cases of cancer worldwide in 2020 (Worldwide cancer data 2022). This disparity points to a significant gap in the provision of palliative care, which is projected to increase with global cancer cases on the rise and the aging of populations worldwide (Palliative care 2020). Currently, there is a consensus on the need for careful evaluation of all aspects of palliative care and more research in this area (Groenvold et al. 2006).

To improve the QoL of patients, information is required about their symptoms and functional problems, which are collected from the patients directly whenever possible (Groenvold et al. 2006). Various questionnaires have been utilized for this task, with the most common being the European Organization for Research and Treatment of Cancer Quality of Life Questionnaire Core 15 for Palliative Care (PAL) (EORTC QLQ-C15-PAL). EORTC QLQ-C15-PAL is the abridged version of the widely used EORTC QLQ-C30. The QLQ-C15-PAL is preferred over its longer counterpart to better suit the ailing palliative population (Golčić et al. 2018). It has also been translated and validated in various languages and populations such as Arabic (Jordan), Croatian, Japanese, Mexican, Spanish, Turkish, and Polish, to name a few (Golčić et al. 2018). Although the QLQ-C30 has been translated and validated in Urdu, the QLQ-C15 has neither been translated nor used in any other form in Pakistan at the writing of this paper (Zahid et al. 2021).

Urdu is the national and official language of Pakistan, the fifth most populous country. While there are more than 10 languages spoken across Pakistan by various ethnic groups, Urdu is understood and spoken throughout. Moreover, a sizeable population of people who understand and speak Urdu reside in India, Bangladesh, Nepal, and countries with a sizable immigrant population from the South Asian region. It is estimated that a total of 230 million people speak Urdu worldwide, making it the 10th most widely spoken language (Zahid et al. 2021; Urdu Language (URD) – L1 & L2 Speakers, Status, Map, Endangered Level & Official Use | Ethnologue Free 2025).

Due to Pakistan being a lower-middle-income country and therefore having a sizable population who are not fluent in English, a palliative questionnaire in English would not be inclusive to patients of a lower sociodemographic standing (Zahid et al. 2021). Additionally, since the QLQ-C15-PAL has not been validated on a Pakistani population, we aim to translate and validate an Urdu QLQ-C15-PAL in an Urdu-speaking population in our study.

## Methods

### Study design

This is an analytical cross-sectional study set at the Aga Khan University Hospital (AKUH), a high-volume, tertiary care center in Karachi, Pakistan. We included patients above the age of 18 admitted to the inpatient palliative care service at AKUH. Patients were not enrolled in the outpatient setting as prior studies conducted at our center have consistently faced challenges with recruitment and data collection in that context (Rafaqat et al. 2023). Patients who were unable to understand or complete the questionnaire, despite assistance from their attendant or nurse, for any reason, were also excluded.

### Sampling technique and sample size calculation

This study utilized non-probability convenience sampling. All eligible patients who consented to participate in the study and completed the questionnaire were included. Self-administration of the questionnaire was encouraged whenever possible, but a registered nurse was present to assist patients if needed. For the sample size calculation, we considered the recommendations of Fidell and Tabachnik, which state that in order to obtain reliable estimates through multivariate analysis, the number of observations should be at least 5 times the number of model variables (Using multivariate statistics, 5th ed.’ 2007; Suárez‐del‐Real et al. 2011). Therefore, a minimum sample size of 75 patients for 15 questionnaire items was identified. Following previous validation studies of EORTC QLQ-C15-PAL, which have sample sizes in the range of 75–150 patients, we decided to adjust our final sample size to 100 patients. All statistical analyses were performed using Stata statistical software (Release 19.5; StataCorp LLC, College Station, TX).

### Data collection tools

Three structured instruments were used in the study: the Urdu-translated version of the EORTC QLQ-C15-PAL, the Edmonton Symptom Assessment System (ESAS), and the Palliative Performance Scale (PPS).

The EORTC QLQ-C15-PAL is a shortened 15-item version of its parent QLQ-C30 questionnaire. The QLQ-C30 has been previously validated in its original English form, as well as in multiple translations, including Urdu (Groenvold et al. 2006; Zahid et al. 2021). The QLQ-C15-PAL comprises of 5 single-item scales (nausea and vomiting, dyspnea, insomnia, appetite loss, and constipation), 2 multi-item functional scales (physical and emotional functioning), 2 multi-item symptom scales (fatigue and pain), and a global health status scale. Responses are recorded on 4- to 7-point Likert scales: a 4-point scale is defined for all items except the 7-point global health scale. Scores are then linearly transformed to a 0–100 scale, where 100 either indicates optimal global health, highest functional status, or worst severity of symptoms, depending on the construct being measured. Scoring was performed according to the EORTC Scoring Manual (Scoring Manual 2017). Before transformation, the questionnaire is designed such that higher scores on items 1 through 14 all imply poorer QoL (in the aspect addressed by that item) but better QoL for item 15. After transformation, high global health scores reflect better QoL, high functional scales indicate better functioning, and high symptom scale scores signify greater symptom burden (i.e., poorer health). Translation of the QLQ-C15-PAL was initiated after receiving permission to proceed from EORTC. The Urdu version of the QLQ-C30 tool, officially shared by the EORTC, was used as a reference. Following translation, the Urdu version of the QLQ-C15-PAL was then submitted to EORTC for approval and is now available in the EORTC Item Library. The complete English and Urdu QLQ-C15-PAL forms are provided in Supplementary Section 1.

The ESAS questionnaire was initially developed by Bruera et al. as a clinical tool to assess the severity of symptoms in patients with advanced cancer (Bruera et al. 1991). Since then, it has been validated in several studies, and its scope has been increased to include the burden of disease in all palliative patient (Bruera et al. 1991; Philip et al. 1998; Chang et al. 2000). ESAS consists of a graphically visualized numerical rating scale (0–10) for 9 specific symptoms including pain, fatigue, nausea, depression, anxiety, drowsiness, appetite, well-being, and shortness of breath. A patient-specific symptom distress score is then calculated by adding the scores for the 9 ESAS items. Patients are asked to rate the symptoms according to the severity experienced in the past 24 h, with higher numbers indicating greater symptom intensity (Yennu et al. 2012).

The PPS, introduced in 1996 as an adaptation of the Karnofsky scale, is an observer-rated tool used to assess performance status in palliative care across 5 domains: Ambulation, Self-care, Activity Level/Evidence of Disease, Intake, and Level of Consciousness. Each domain is scored in increments of 10% from 0% to 100% (Baik et al. 2018). It has been widely translated, validated, and applied (Ho et al. 2008). Patients were classified into an early-integration and late-integration group based on their score on the PPS.

### Data collection

Patients who may benefit from palliative care at our institute are referred to the service by their primary team regardless of disease stage or treatment intent. The palliative care team consists of specialized physicians and nurses, supported by consultants from other specialties.

Following completion of the initial clinical evaluation by the team, patients were offered participation in our study. Nurses obtained informed consent and patients willing to participate were asked to self-administer the included questionnaires in their presence. Patients unable to fill out the forms independently were supported by their attendants, who were asked to read out the questions to the patients and document their responses verbatim without leading or prompting. In the absence of attendants, assistance was provided by the nurses. Once data collection was complete, all identifiers were removed to maintain the privacy of the participants. The data will be retained for 7 years as per institutional policy.

### Statistical analysis

Prior to conducting statistical analyses, the data was cleaned on Microsoft Excel and cases with missing data were excluded. All statistical analyses were performed using Stata (release 19.5). Categorical variables were reported as frequencies, while continuous variables were expressed as means with standard deviations or medians with interquartile ranges. Cronbach’s alpha was calculated to assess reliability of the overall questionnaires and/or subscales. The construct (convergent and divergent) validity of scales and items was tested using Pearson correlations between item raw scores and subscale or item transformed scores. Previous studies have considered alphas >0.7 to indicate acceptable reliability, and correlation coefficients >0.4 and <−0.4 to indicate convergent and divergent validity respectively (Arraras et al. 2014; Miyashita et al. 2015). We do not feel that there are clear cutoffs that cleanly indicate the presence or absence of reliability or validity, but readers may keep these values in mind. Correlation coefficients between transformed subscale scores, and between the results of the ESAS and PPS, were also determined and reported. Known-group validity was evaluated by comparing domain scores across high (≥40) versus low (≤30) PPS subgroups. All *p*-values were 2-tailed and those <0.05 were considered statistically significant.

## Results

### Participant characteristics

A total of 105 patients consented to participate, of whom 100 completed the questionnaire and were included in the study. The sample included 56 men and 44 women with a mean age of 57.8 years (±14.8). The mean PPS score was 36.2 (±17.3) and 38 patients had a known metastatic disease. Treatment modalities included surgery (*N* = 49), chemotherapy (*N* = 65), and radiation (*N* = 39) and were curative-intent in 59 participants. Four patients were able to fill out the questionnaire independently, while the rest required assistance from attendants or nurses. Additional patient characteristics are described in Table 1.

**Table 1.** Patient characteristics (*N* = 100)

Variable	*n*
Age	
Mean (SD)	57.8 (14.8)
Median (IQR)	58.5 (47.5–69.0)
*Sex*	
Male	56
Female	44
*Primary site of disease*^a^	
Gastrointestinal	21
Breast	17
Head and neck	17
Genitourinary	12
Lung	11
Liver	6
Unknown primary	5
Hematologic	4
Musculoskeletal/Other	4
CNS	3
*Metastasis*	
Yes	38
No	62
Palliative Performance Scale	
Mean (SD)	36.2 (17.3)
Median (IQR)	40 (20–50)
≤30	47
≥40	53
*Treatment intent*	
Curative	59
Palliative	14
None ^b^	27
*Treatment modality*	
Surgery	49
Chemotherapy	65
Radiotherapy	39
*Questionnaire completion*	
Patient	4
Healthcare professional assisted	41
Family caregiver assisted	52
Paid caregiver assisted	3

a Primary tumor sites were categorized based on anatomical and clinical relevance to enhance interpretability in the context of palliative care. The “Gastrointestinal” group includes esophagus, stomach, rectum, liver, gallbladder, pancreas, and peritoneum. The “Genitourinary” group includes the bladder, kidney, ovaries, cervix, and vagina. The “Head and neck” group includes the buccal cavity, tongue, nasopharynx, and oropharynx. The “Musculoskeletal/Other” group includes bone, hand, and chest wall. Sites of unknown origin were categorized as “Unknown primary.”

b Not receiving anticancer therapy; symptomatic management and supportive care only.

**Figure 1.**
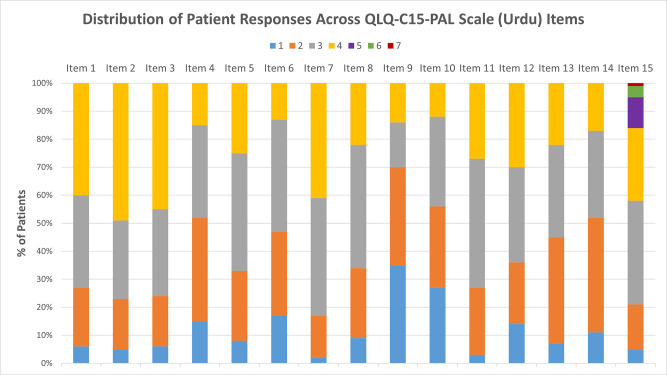
Distribution of patient responses for each questionnaire item. All items were scored on a scale of 1–4 with the exception of item 15, which was scored from 1 to 7. A higher score indicates a worse quality of life.

The distributions of raw scores for all QLQ-C15-PAL items, which directly correspond with Likert scale responses that patients could select, are reported in Figure 1. After linear transformation, the mean global QoL score was 39.7 (20.1), while mean physical and emotional function scores were 28.6 (27.9) and 46.0 (27.9), respectively. Mean physical symptom scores ranged from 36.3 (34.5) for nausea to 59.7 (29.7) for anorexia, while those for pain and fatigue were 60.7 (29.4) and 69.8 (22.9), respectively (Table 2). The distribution of ESAS item scores is provided in Table 3.

**Table 2.** Cronbach’s alpha coefficients and the mean scores^a^ for each subscale/item (*N* = 100)

EORTC QLQ-C15-PAL (version 1)	Mean (SD)	Cronbach’s alpha coefficient
*Physical function*	28.6 (27.9)	**0.893**
Do you have any trouble taking a short walk outside of the house?		
Do you need to stay in bed or in a chair during the day?		
Do you need help with eating, dressing, washing yourself, or using the toilet?		
*Pain*	60.7 (29.4)	**0.805**
Have you had pain?		
Did pain interfere with your daily activities?		
*Fatigue*	69.8 (22.9)	0.697^b^
Have you felt weak?		
Were you tired?		
*Physical symptoms*		
Were you short of breath?	49.3 (30.9)	
Have you had trouble sleeping?	49.7 (30.9)	
Have you lacked an appetite?	59.7 (29.7)	
Have you felt nauseated?	36.3 (34.5)	
Have you been constipated?	43.0 (33.3)	
*Emotional function*	46.0 (27.9)	**0.851**
Did you feel tense?		
Did you feel depressed?		
*Global health status/Quality of life*	39.7 (20.1)	
How would you rate your overall quality of life during the past week?		
**Overall questionnaire**		**0.862**^c^

a Mean scores are linearly transformed to a standardized scale ranging from 0 to 100. Higher quality of life or function scores indicate better quality of life or function, while higher symptom scores indicate more severe symptoms.

b The fatigue subscale result did not meet the prespecified criterion of 0.7. All other values exceeded the prespecified criterion. All other values **(bold)** exceeded this criterion.

c The overall Cronbach’s alpha was computed by calculating and pooling standardized scores on a 0–100 scale for all 15 items individually.

**Table 3.** Mean scores^a^ for each subscale/item of the ESAS-r (*N* = 100)

ESAS-r symptom	Mean (SD)
Pain	3.9 (2.5)
Tiredness	4.4 (2.5)
Drowsiness	2.4 (2.9)
Nausea	1.7 (2.0)
Lack of appetite	3.7 (2.5)
Shortness of breath	3.1 (2.7)
Depression	3.1 (2.5)
Anxiety	3.1 (2.5)
Well-being	5.0 (2.4)
Other problem^b^	3.1 (2.4)

a Each item in the ESAS is scored on a scale of 0–10 with higher scores indicating worse symptomatology/well-being/quality of life.

b ‘Other problems’ were specified by 33 patients and included constipation, abdominal distension, diarrhea, vomiting, seizures, insomnia, weakness, lower limb edema, wound infection, coughing, and blood in sputum.

### Validation

Cronbach’s alpha for the entire questionnaire was 0.862. All subscales achieved coefficients >0.7 (Table 2) except fatigue, which had a Cronbach’s alpha of 0.697 (Thorndike 1995).

Pearson correlations between item raw scores and item or subscale transformed scores are reported in Table 4, while those between subscale transformed scores are reported in Table 5. For convergent validity, all correlation coefficients between item raw scores and related subscale transformed scores exceeded 0.8 (absolute values, range: 0.872–0.934; green boxes, Table 4). Of the 112 correlations between “unrelated” items and subscales that were used to assess divergent validity (i.e., excluding correlations between items and the subscales derived from them, items and themselves, and between items and global QoL which is “related” to all items), only 15 (13%) had absolute values >0.4, the largest of which was 0.519 (orange boxes, Table 4) between items pertaining to insomnia and the incidence of pain that interfered with daily activities, respectively. Similar results were seen in the correlations between transformed subscale scores (Table 5).

**Table 4.** Pearson’s correlation coefficients between the items and subscales in EORTC the QLQ-C15-PAL^a^ (*N* = 100)

	Subscales									
Item	Physical functioning (items 1–3)	Pain (items 5, 12)	Fatigue (items 7, 11)	Emotional functioning (items 13, 14)	Dyspnea (item 4)	Insomnia (item 6)	Lack of appetite (item 8)	Nausea (item 9)	Constipation (item 10)	Global quality of life (item 15)
1. Do you have any trouble taking a short walk outside of the house?	−0.911*	0.345	0.458^#^	−0.328	0.303	0.373	0.325	0.141	0.120	−0.433
2. Do you need to stay in bed or a chair during the day?	−0.913*	0.305	0.330	−0.244	0.202	0.295	0.241	0.044	−0.045	−0.432
3. Do you need help with eating, dressing, washing yourself, or using the toilet?	−0.899*	0.343	0.390	−0.380	0.163	0.279	0.149	0.049	0.040	−0.487
7. Have you felt weak?	−0.357	0.370	0.872*	−0.419^#^	0.317	0.342	0.434^#^	0.266	0.257	−0.351
11. Were you tired?	−0.401^#^	0.388	0.880*	−0.444^#^	0.375	0.362	0.446^#^	0.273	0.240	−0.483
5. Have you had pain?	−0.260	0.904*	0.295	−0.349	−0.016	0.351	0.147	0.125	0.335	−0.299
12. Did pain interfere with your daily activities?	−0.399	0.928*	0.486	−0.462^#^	0.134	0.519^#^	0.274	0.350	0.294	−0.429
13. Did you feel tense?	−0.374	0.509^#^	0.491^#^	−0.932*	0.273	0.375	0.161	0.204	0.303	−0.353
14. Did you feel depressed?	−0.280	0.327	0.429^#^	−0.934*	0.182	0.296	0.130	0.196	0.295	−0.302
4. Were you short of breath?	−0.245	0.070	0.395	−0.244	1.000	0.312	0.123	0.018	0.088	−0.238
6. Have you had trouble sleeping?	−0.348	0.481^#^	0.402^#^	−0.359	0.312	1.000	0.187	0.248	0.282	−0.286
8. Have you lacked appetite?	−0.262	0.235	0.503	−0.156	0.123	0.187	1.000	0.470^#^	0.171	−0.358
9. Have you felt nauseated?	−0.086	0.267	0.307	−0.214	0.018	0.248	0.470^#^	1.000	0.092	−0.109
10. Have you been constipated?	−0.043	0.342	0.283	−0.320	0.088	0.282	0.171	0.092	1.000	−0.202
15. How would you rate your overall quality of life during the past week?	0.496	−0.402	−0.477	0.351	−0.238	−0.286	−0.358	−0.109	−0.202	1.000

a Raw scores (0–4 for items 1–14; 0–7 for item 15) for each item were correlated with corresponding subscale scores linearly transformed to a standardized 0–100 scale.

* boxes indicate correlation coefficients used to gauge convergent validity.

# boxes indicate correlation coefficients used to gauge discriminant/divergent validity that had an absolute value >0.4.

**Table 5.** Pearson’s correlation coefficients between the subscales of EORTC QLQ-C15 PAL^a^ (*N* = 100)

Subscales	Physical functioning	Pain	Fatigue	Emotional functioning	Physical symptoms
Physical function (1–3)	1.000				
Pain (5, 12)	−0.365	1.000			
Fatigue (7, 11)	−0.433	0.433	1.000		
Emotional (13, 14)	0.350	−0.447	−0.492	1.000	
Physical symptoms (4, 6, 8–10)	−0.320	0.467	0.625	−0.433	1.000

a Correlations among the subscales of the EORTC QLQ-C15-PAL were assessed using linearly transformed scores standardized to a 0–100 scale.

Concurrent validity was assessed by correlating transformed item/subscale scores on the EORTC QLQ-C15-PAL with ESAS scores (Table 6). Related scores across the EORTC/ESAS forms had weaker correlations than those seen in the analysis of convergent validity. The strongest one observed was between dyspnea on the QLQ-C15-PAL and shortness of breath on the ESAS, which had a correlation coefficient of 0.579.

**Table 6.** Pearson’s correlation coefficients between the items of EORTC QLQ-C15-PAL and ESAS^a^ (*N* = 100)

	ESAS								
QLQ-C15-PAL Scale/Item	Pain	Tiredness	Drowsiness	Nausea	Lack of appetite	Shortness of breath	Depression	Anxiety	Well-being
Physical functioning	−0.207	−0.380	−0.374	0.120	−0.095	−0.153	−0.229	−0.246	−0.303
Pain	0.547	0.214	0.071	0.086	0.044	−0.061	0.091	0.112	0.186
Fatigue	0.282	0.472	0.267	0.127	0.222	0.196	0.230	0.294	0.246
Emotional functioning	−0.259	−0.320	−0.034	−0.094	−0.003	−0.066	−0.524	−0.345	−0.249
Dyspnea	0.105	0.191	0.096	−0.015	0.064	0.579	0.271	0.264	0.246
Insomnia	0.295	0.057	0.046	−0.029	−0.008	0.070	0.209	0.182	0.237
Loss of appetite	0.206	0.237	0.302	0.227	0.546	−0.025	0.038	0.045	0.071
Nausea and vomiting	0.125	0.133	0.033	0.544	0.347	−0.051	0.034	−0.105	−0.174
Constipation	0.286	0.046	0.051	−0.026	−0.072	−0.028	0.145	0.157	0.188
Global quality of life	−0.157	−0.295	−0.290	−0.063	−0.223	−0.101	−0.237	−0.141	−0.151

a Linearly transformed QLQ-C15-PAL domain scores (0–100 scale) were correlated with corresponding ESAS scales.

In the analysis of known-group validity, patients with higher PPS scores (≥40) had superior global quality of life, physical and emotional functioning, and reduced fatigue and anorexia (all *p* < 0.05). No evidence for superior quality of life in the low PPS score group was observed in any QLQ-C15-PAL subscale or domain (Table 7).

**Table 7.** Statistical analysis of the known-group validity; comparing EORTC QLQ-C15-PAL scores^a^ (*N* = 100)

QLQ-C15-PAL Scales	PPS categories		*t*	*p*
	≤30 (*N* = 47)	≥40 (*N* = 53)		
	Mean score (SD)			
Physical functioning	12.5 (18.9)	42.8 (26.9)	−6.56	**<0.001**
Pain	65.2 (29.0)	56.6 (29.3)	1.48	0.142
Fatigue	77.0 (21.0)	63.5 (22.9)	3.06	**0.003**
Emotional functioning	39.7 (24.5)	51.6 (29.8)	−2.18	**0.032**
Dyspnea	52.5 (32.4)	46.5 (29.5)	0.95	0.342
Insomnia	54.6 (29.0)	45.3 (32.1)	1.53	0.130
Loss of appetite	66.7 (28.7)	53.5 (29.5)	2.27	**0.026**
Nausea and vomiting	36.2 (36.0)	36.5 (33.5)	−0.04	0.965
Constipation	45.4 (32.2)	40.9 (34.4)	0.68	0.500
Global quality of life	31.6 (20.6)	46.9 (16.7)	−4.04	**<0.001**

a Mean scores are linearly transformed to a standardized scale ranging from 0 to 100; higher scores indicate greater symptom severity/better functioning, depending on the domain. **Bold** indicates a statistically significant result (*p*<0.05).

## Discussion

The EORTC QLQ-C15-PAL is a tool used to assess the QoL of cancer patients receiving palliative care and has been translated and validated in various languages (Arraras et al. 2014; Miyashita et al. 2015; Ozcelik et al. 2016). Our study translated the EORTC QLQ-C15-PAL into Urdu and established its reliability and validity across multiple indices. Urdu is the national language of Pakistan and the tenth most commonly spoken language globally (Ambreen and To 2025). Therefore, this work will facilitate clinicians and researchers around the world with a useful and familiar tool to measure self-reported QoL in this population.

Based on previously published translations of the tool, our work demonstrates that the Urdu version of the EORTC QLQ-C15-PAL is reliable and valid (Oñate-Ocaña et al. 2009; Huijer et al. 2013; Arraras et al. 2014; Miyashita et al. 2015; Ozcelik et al. 2016). All Cronbach’s alpha coefficients were well in excess of the prespecified reliability threshold (>0.7) with the exception of the fatigue domain, for which the coefficient fell short of this criterion at 3 significant figures. This suggests that the tool is internally consistent. Strong correlations were seen between individual form items and the relevant subscales derived from them (all *r* > 0.8), indicating convergent validity, while a uniform absence of these between “unrelated” domains supports the discriminant validity of the questionnaire. These statistics show that questions about the same facet of QoL overlap and are likely to measure the same subjective aspect of patients’ health while also providing distinct information from other, “unrelated” questions pertaining to different domains. Finally, known-group validity was established as both improved function and decreased symptomatology were observed in patients with high PPS across several domains. This helps to support the tool’s validity by demonstrating its ability to identify poorer QoL in a sicker subgroup of patients.

Our assessment of concurrent validity was less informative as only modest correlations were observed between domains where strong associations were expected. This could be the result of several differences in the design and administration of these questionnaires. Although both the ESAS and QLQ-C15-PAL assess QoL in palliative settings, the ESAS is a rapid bedside assessment which can vary based on patients’ conditions at different times while the QLQ-C15-PAL evaluates multiple domains of QoL over the preceding week (Harsono et al. 2022). The premise of translating and validating questionnaires in patients’ preferred languages is that differences in self-reported outcomes may arise due to language barriers. We believe that this may also account for the lack of strong concordance between responses to the 2 forms.

Our study sample was formed by a near equal balance of both male and female patients and included younger adults as well as geriatric patients. Furthermore, a mix of cancers originating from multiple organ systems including the gastrointestinal tract, breast, head and neck, genitourinary system, and lungs was present. Patients with localized and metastatic cancer undergoing multimodal therapy with curative and palliative treatment-intent were all represented. Thus, we believe the translated tool is generally applicable. However, it must be noted that, due to logistical constraints that have resulted in poor data quality in the past, we did not include patients from the outpatient setting and our sample may thus represent a “sicker” subset of cancer patients. Conversely, at AKUH, we implement a strategy where palliative care is involved early in patients’ courses of treatment, and this may have contributed to the high representation of patients with localized cancer who were receiving curative-intent therapy. In addition, this was a single-center study at a private hospital that does not offer free-of-charge care to most patients. Thus, replication studies at public settings elsewhere are warranted. In addition, although Urdu is widely spoken throughout the country, Pakistan is home to many communities who primarily speak regional languages like Sindhi, Punjabi, Pashto, and others (Language data for Pakistan 2023). Ensuring that these patients do not experience suboptimal care or discrimination due to language barriers is crucial, and translations of QoL tools into these languages may help to prevent such inequity. Although some degree of responder bias is likely to have affected our results, we allowed patients to receive assistance from attendants or research staff when filling out our form to ensure frail patients were not excluded. Although this may have led to some distortion of “self”-reported QoL, we felt that this approach was pragmatic and more representative of how such tools are used in routine practice.

## Conclusion

To conclude, the Urdu version of the EORTC QLQ-C15-PAL is now available, and is a reliable and valid tool to measure self-reported QoL in cancer patients who speak Urdu. Future studies are needed to replicate our results at other sites in Pakistan and abroad, as well as in outpatient settings. Furthermore, we advocate for the inclusion of patient-reported outcomes as endpoints in clinical research and for the representation of diverse patients in study samples. Our tool enables researchers to enroll patients who speak Urdu in such studies.

## Supporting information

10.1017/S1478951525101521.sm001 Jamil et al. supplementary material Jamil et al. supplementary material

## Supplementary material

The supplementary material for this article can be found at https://doi.org/10.1017/S1478951525101521.
