# Supplementary material for: Bridging the language gap in palliative oncology: A translation and validation of the Urdu version of the EORTC QLQ-C15-PAL
Source: Palliat Support Care. 2026 Jan 21;24:e41. doi: 10.1017/S1478951525101521 (PMC13166730; doi:10.1017/S1478951525101521)

Supplementary Material

**Bridging the Language Gap in Palliative Oncology: A Translation and Validation of the Urdu version of the EORTC QLQ-C15-PAL**

M. Abdullah Jamil^1^, Syed Balaj Ali Rizvi^1^, Aisha Ambreen^2^, Asra Taj^2^, Ismat Jabeen^2^, Habiba Zaheer^2^, Hunza Asher^2^, Mahnoor Javed^1^, Omar Mahmud^1^, Muhammad Atif Waqar^2^

^1^ Medical College, Aga Khan University, Karachi 74800, Pakistan.

^2^ Section of Palliative Medicine, Department of Oncology, Aga Khan University Hospital, Karachi 74800, Pakistan.

**Corresponding Author:**

Muhammad Atif Waqar, MD

Section of Palliative Medicine,

Department of Oncology,

Aga Khan University Hospital,

Karachi 74800, Pakistan.

**Email:** [atif.waqar@aku.edu](mailto:atif.waqar@aku.edu)

**Section 1: Questionnaires**

**EORTC QLQ-C15 PAL: English Version**


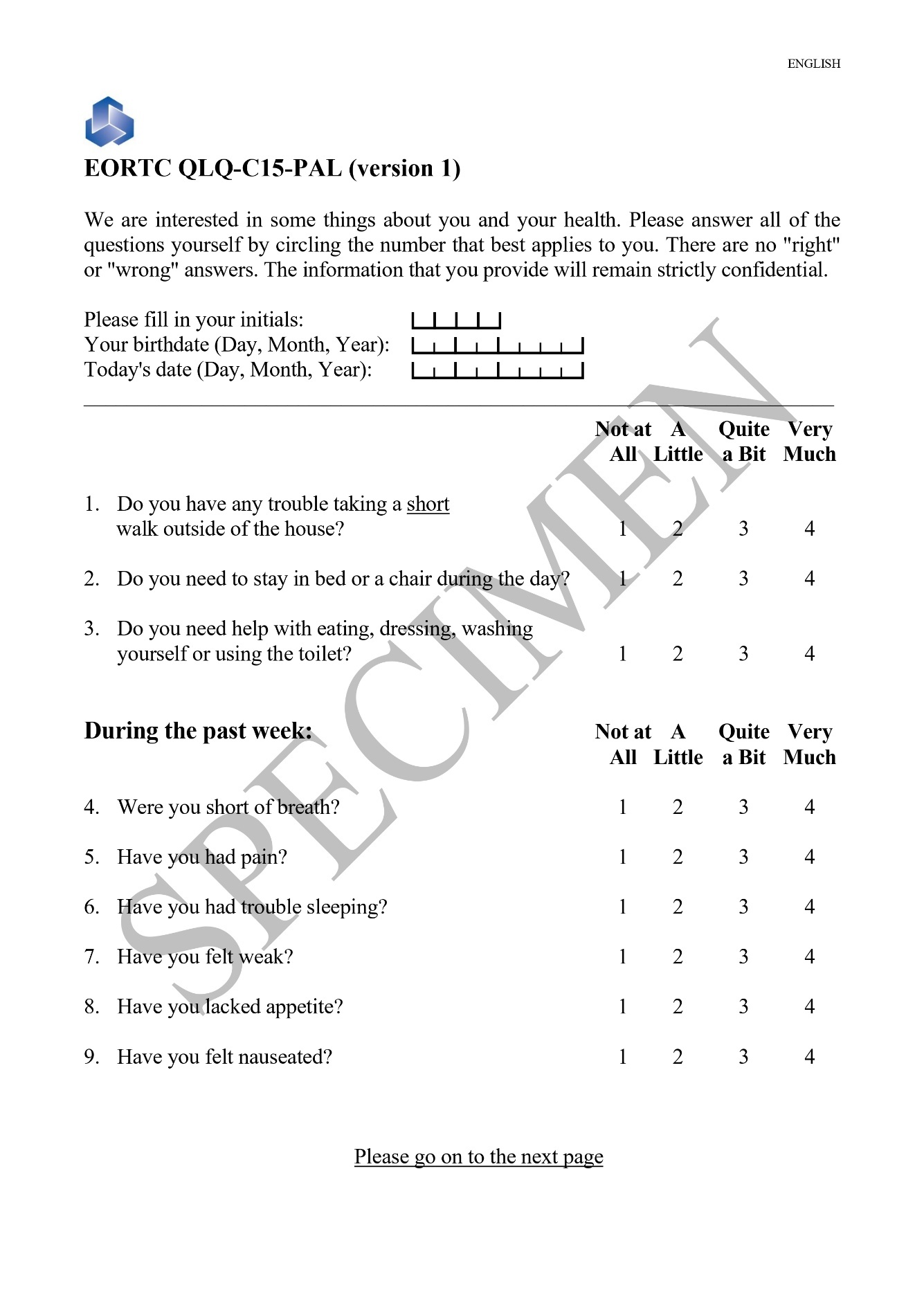


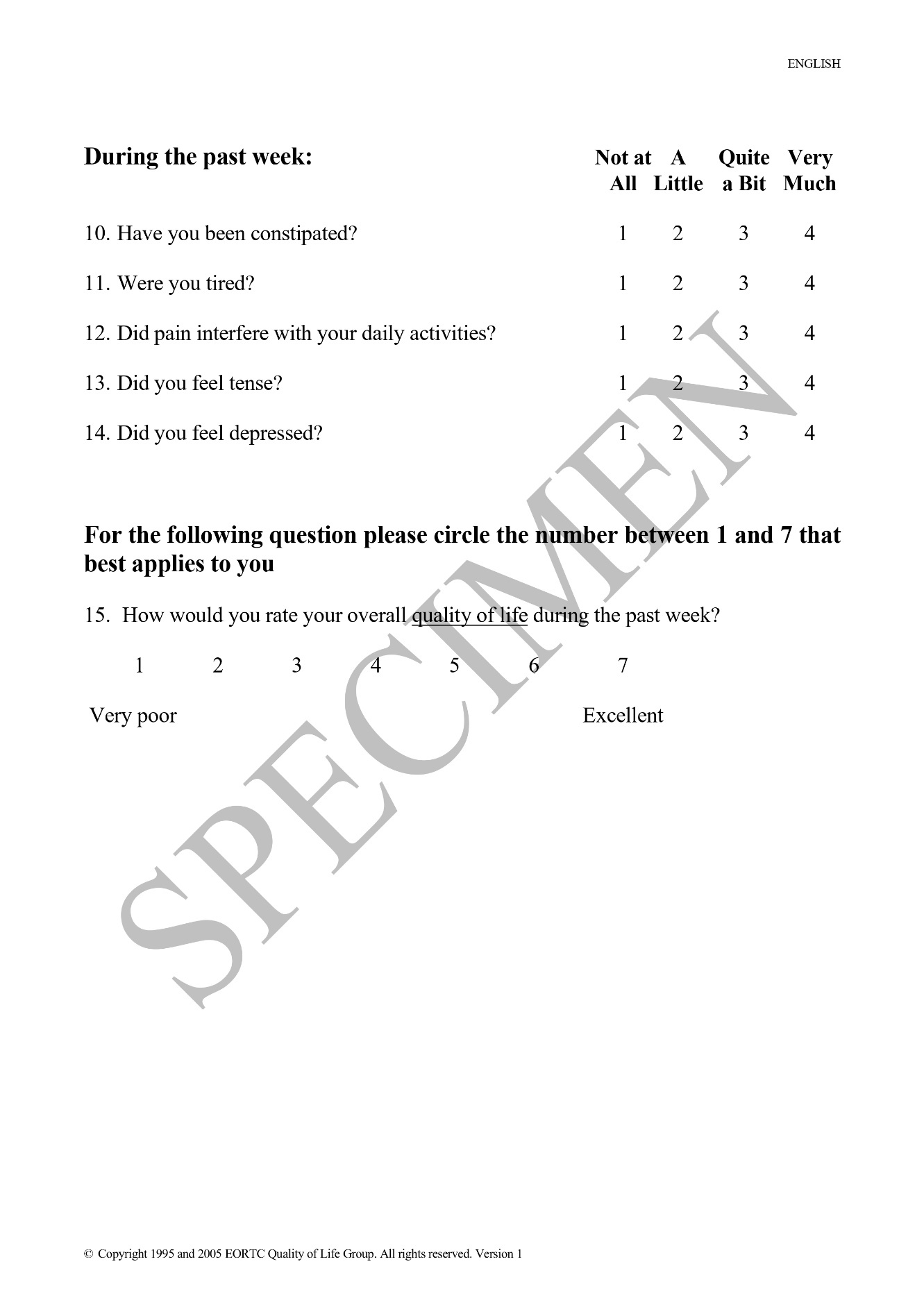


**EORTIC QLQ-C15 PAL: Urdu Version**


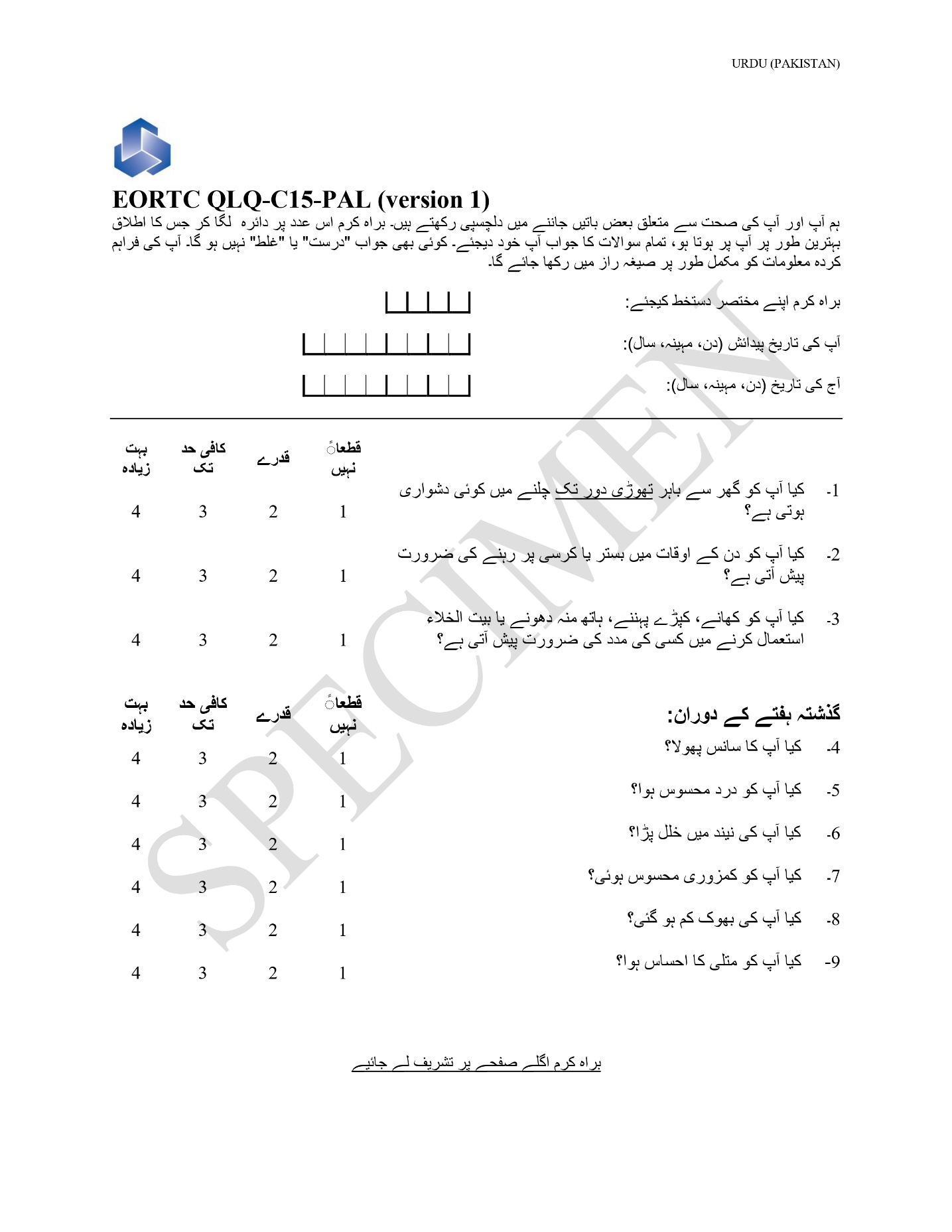


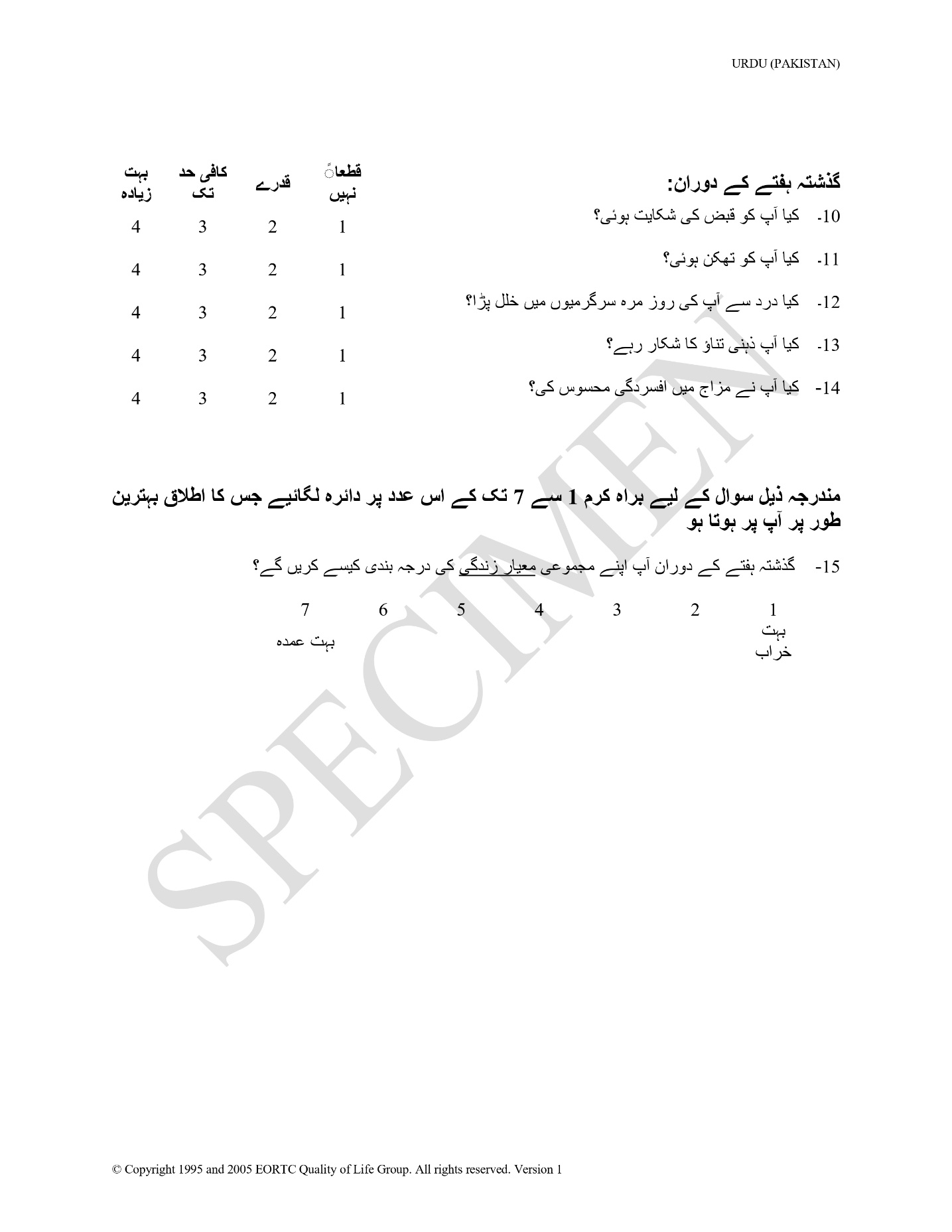

Supplement: Jamil et al. supplementary material [file S1478951525101521sup001.docx]
